# Sex-specific patterns of gene expression following influenza vaccination

**DOI:** 10.1038/s41598-018-31999-x

**Published:** 2018-09-10

**Authors:** Feng Wen, Jinyue Guo, Zhili Li, Shujian Huang

**Affiliations:** College of Life Science and Engineering, Foshan University, Foshan, 528231 Guangdong China

## Abstract

Sex-based variations in the immune response to the influenza vaccines was reported, however, the genetic basis responsible for the sex variations in the immune response toward the influenza vaccines remains unclear. Here, the genes responsible for sex-specific responses after vaccination with trivalent inactivated influenza virus were identified. These genes were enriched in virus response pathways, especially interferon signaling. A list of genes showing different responses to the vaccine between females and males were obtained next. Our results demonstrated that females generate stronger immune responses to seasonal influenza vaccines within 24 hours than males. However, most of these genes with variability between sexes had the opposite expression levels after three days, suggesting that males retained the immune responses longer than female. To summary, our study identified genes responsible for the sex variations toward influenza vaccination. Our findings might provide insights into the development of the sex-dependent influenza vaccines.

## Introduction

Seasonal epidemics of influenza affect 2–7% of the population annually. As reported by the World Health Organization (WHO), seasonal influenza is responsible for up to half a million deaths and nearly 5 million hospitalizations worldwide^1^. As early as 1933, humans have started to study immunization pre-dated virus^2^. Even today, vaccination remains the cornerstone of influenza prevention strategies. To date, there are two main types of commercial licensed vaccines and two classes of antivirals that are effective against influenza. According to animal models, it was reported that immune responses were strongly influenced by host genetic factors^3^. However, the details of how humans respond to vaccination, such as variability caused by host genetic factors, remain unclear.

One of the known factors for inter-individual variability is sex. For example, immunological responses to influenza virus vaccines was found to differ between males and females^4^. Immune responses were consistently hi jigher in adults, particularly elderly women, rather than men of comparable ages. Interestingly, it has been reported that the males had higher incidence of infection to seasonal influenza viruses than females in Spain and the United States of America^5,6^. Pulcini *et al*. reported that females are less likely to develop strong antibody response to influenza vaccines as males did^7^. The protective antibody response for viral vaccines in females can be two timers higher than that in males^8^. Animal studies suggested female mice of reproductive age generate higher antibody titer and are better protected from lethal heterosubtypic influenza strain than do male mice^9^. It has been reported that the outcome of influenza is worse in males than females younger than 18 years of age^10^. Quandelacy *et al*. showed that the influenza-associated mortality rate was higher in adult male than adult female^11^. However, pregnant woman and females over the age of 65 reported more serve adverse reactions to influenza vaccines than their counterparts^12–16^.

Franco, *et al*.^1^ and Bucasas, *et al*.^17^ measured gene transcription and antibody responses to influenza vaccination and identified gene sets correlated with the antibody response. They immunized 119 healthy adult male volunteers with the trivalent influenza vaccine, which is typically composed of three virus strains that have been inactivated and partially purified. An independent group including 128 ethnically homogeneous healthy adult female volunteer was used as a validation. Both the males and females were aged from 18 to 40 and were self-reported as Caucasian. However, the identification of genetic differences between the males and females to influenza vaccines is still lacking. In this study, we used bioinformatics strategies to determine the genetic basis of sex-specific differences after influenza vaccination.

## Results

### Differentially expressed genes shared by male and female

**Figure 1:**
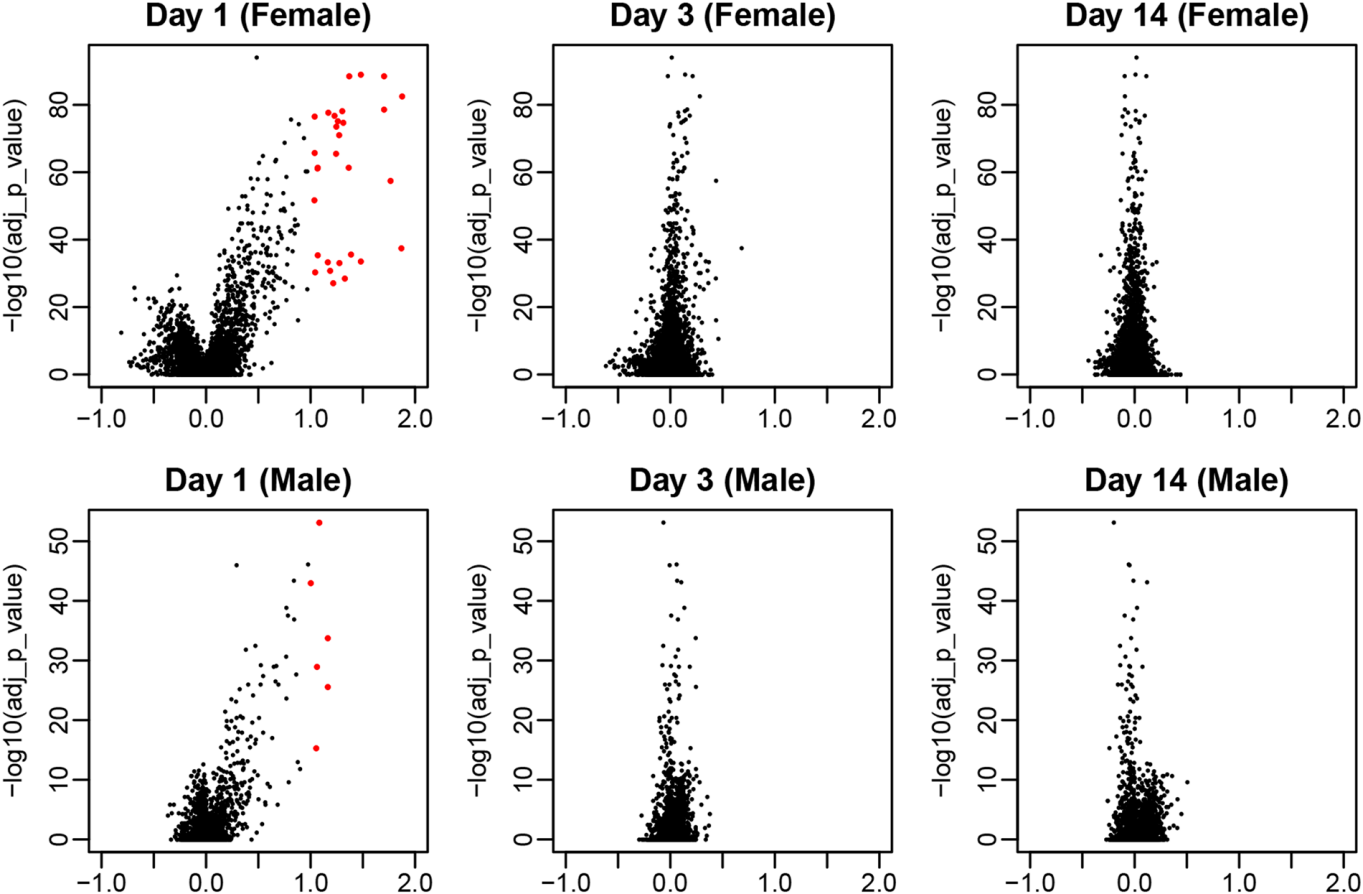
The volcano plot of the differentially expressed genes in males and females compared with the baseline (Day 1, Day 3, and Day 14). The genes with significantly altered expression are annotated as red. The cutoff was set so that the FDR (false discovery rate) was lower than 0.01 and log fold change was larger than 1. The x-axis represents the log fold change relative to baseline.

The genes that were up-regulated or down-regulated in post-vaccination Day 1, Day 3 or Day 14 compared with the baseline for whole blood RNA samples collected from females and male were identified separately. There were 30 and 6 significantly differentially expressed genes for female and male, respectively (Fig. 1). Data were analyzed using the ANOVA model, and was compared to the baseline set for each time point, Day 1, 3, or 14 post-vaccination. The cut off was set so that false discovery rate (FDR) was lower than 0.01 and log2 fold changes were considered, only if they were larger than 1 (Fig. 1).

All of the differentially expressed genes were observed on Day 1 in both females and males, whereas no genes demonstrated changes in expression on Day 3 or Day 14. When considering only the FDR, more genes were significantly regulated on Day 1, than either Day 3 or Day 14. These results suggested that the immune response to vaccination occurred within the first 24 hours and returned to the baseline within 3 days.

**Figure 2:**
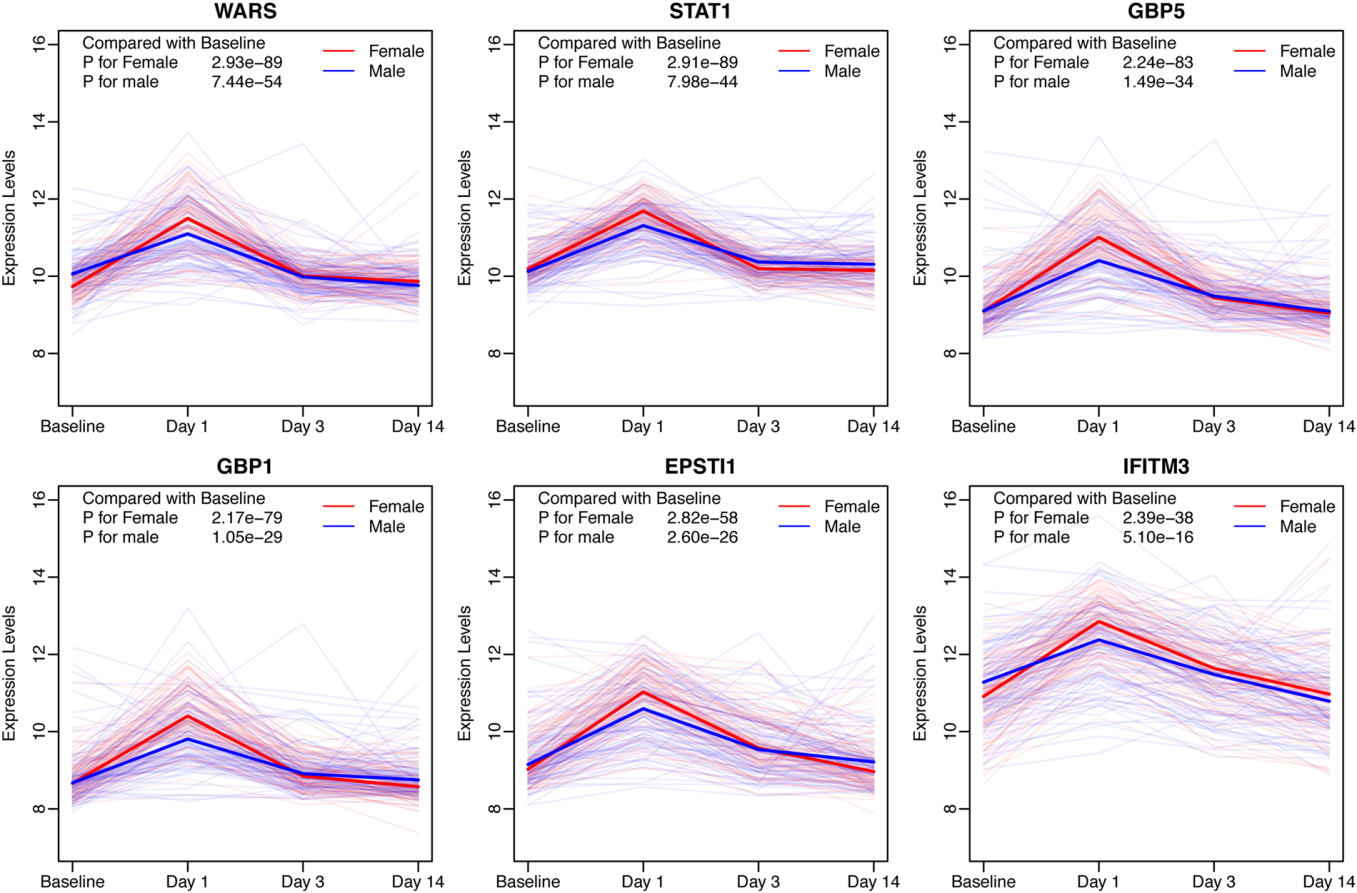
The response over time profiles of activated genes (*WARS*, *STAT1*, *GBP5*, *GBP1*, *EPSTI1*, and *IFITM3*) shared by females and males in response to vaccination. The gene expression levels of females and males are shown as red and blue, respectively. The solid lines represented the median in the two groups for each gene.

Moreover, all six genes that were differentially expressed in males, were also differentially expressed in females, listed in Fig. 2. Most of the genes identified have been reported to act in the immune system except for *WARS* (Table 1). Analysis of the response over time of these six genes showed that they were all up-regulated on Day 1 and almost recovered to the baseline by Day 3 (Fig. 2).

**Table 1:** The activated genes after influenza vaccination shared in both females and males.

	Female		Male		Full Name	Effects
	P Value	FDR	P Value	FDR		
WARS	1.2E-93	2.9E-89	3.0E-58	7.4E-54	tryptophanyl-tRNA synthetase	NA
STAT1	1.2E-93	2.9E-89	3.2E-48	8.0E-44	signal transducer and activator of transcription 1	response to virus
GBP5	8.9E-88	2.2E-83	5.9E-39	1.5E-34	guanylate binding protein 5	inflammatory response
GBP1	8.6E-84	2.2E-79	4.2E-34	1.1E-29	guanylate binding protein 1	response to virus
EPSTI1	1.1E-62	2.8E-58	1.0E-30	2.6E-26	epithelial stromal interaction 1	enhanced by vaccine
IFITM3	9.5E-43	2.4E-38	2.0E-20	5.1E-16	interferon induced transmembrane protein 3	response to virus

We also lowered the cutoff for the differentially expressed genes by removing the requirements for fold change and thus to identify the biological functions of the differentially expressed genes shared by males and females. As expected, these genes were enriched in pathways that correspond to host responses to viruses. Notably, interferon signaling pathways seemed to be extremely important in the response to influenza vaccination.

### Variability in gene expression between females and males

More genes were identified differentially expressed after vaccination in females than males. Furthermore, these differentially expressed genes demonstrated larger changes in expression in females. This suggested that females might generate stronger immune responses to seasonal influenza vaccines, which might lead to enhanced vaccine efficacy.

**Figure 3:**
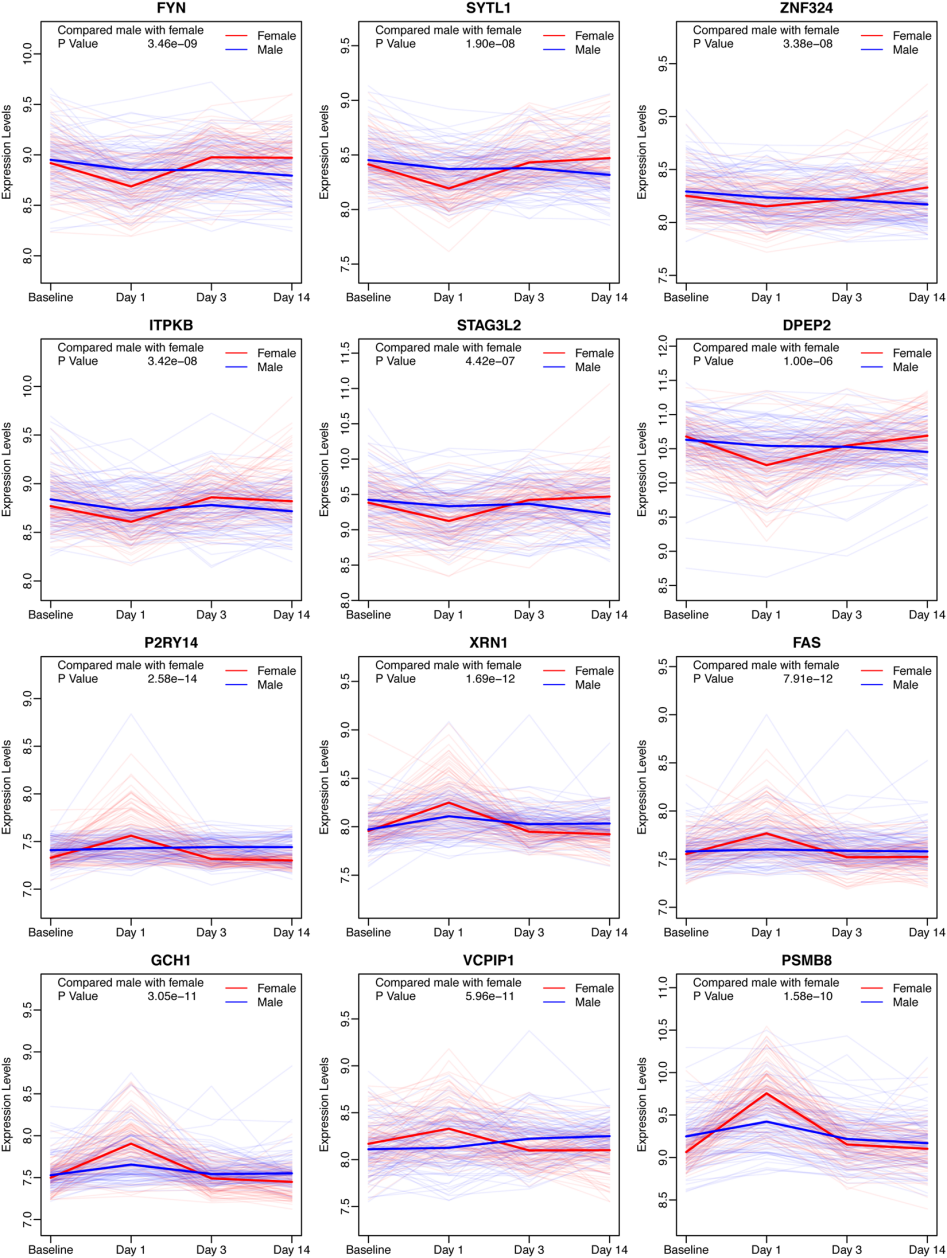
The genes with significantly varied expression levels between females and males. The genes in the top two rows of panels were expressed lower in females on Day 1, and the genes in the lower two rows of panels were highly expressed in females on Day 1, which represented two different patterns of these differentially expressed genes.

To further study the variability of responses to the influenza vaccines, the genes that were differentially expressed between females and males after administration of the vaccine were selected. After removing the genes that originally showed differences on the baseline, these remaining genes showed two patterns, for which representative examples are shown (Fig. 3). A total of 182 genes were more highly expressed in females than males, and an additional 88 genes were expressed lower in females, 24 hours after vaccination. Interestingly, many of these genes were also differentially expressed, in the opposing direction, three days post-vaccination, and almost half of the genes remained the same as Day 3, 14 days after vaccination. The detailed counts were listed as Table 2A,B.

**Table 2:** The comparison of numbers of differentially expressed genes between Day 3 and Day 14 post the influenza vaccination.

		Day 3		
		Lower in Female	Same	Higher in Female
**A**				
Day 14	Lower in Female	87	5	0
	Same	87	2	1
	Higher in Female	0	0	0
**B**				
Day 14	Lower in Female	0	0	0
	Same	0	0	51
	Higher in Female	0	4	33

(A) The number of genes with lower expression levels in females than in males on Day 3 and Day 14. (B) The number of genes with higher expression levels in females than in males on Day 3 and Day 14.

**Figure 4:**
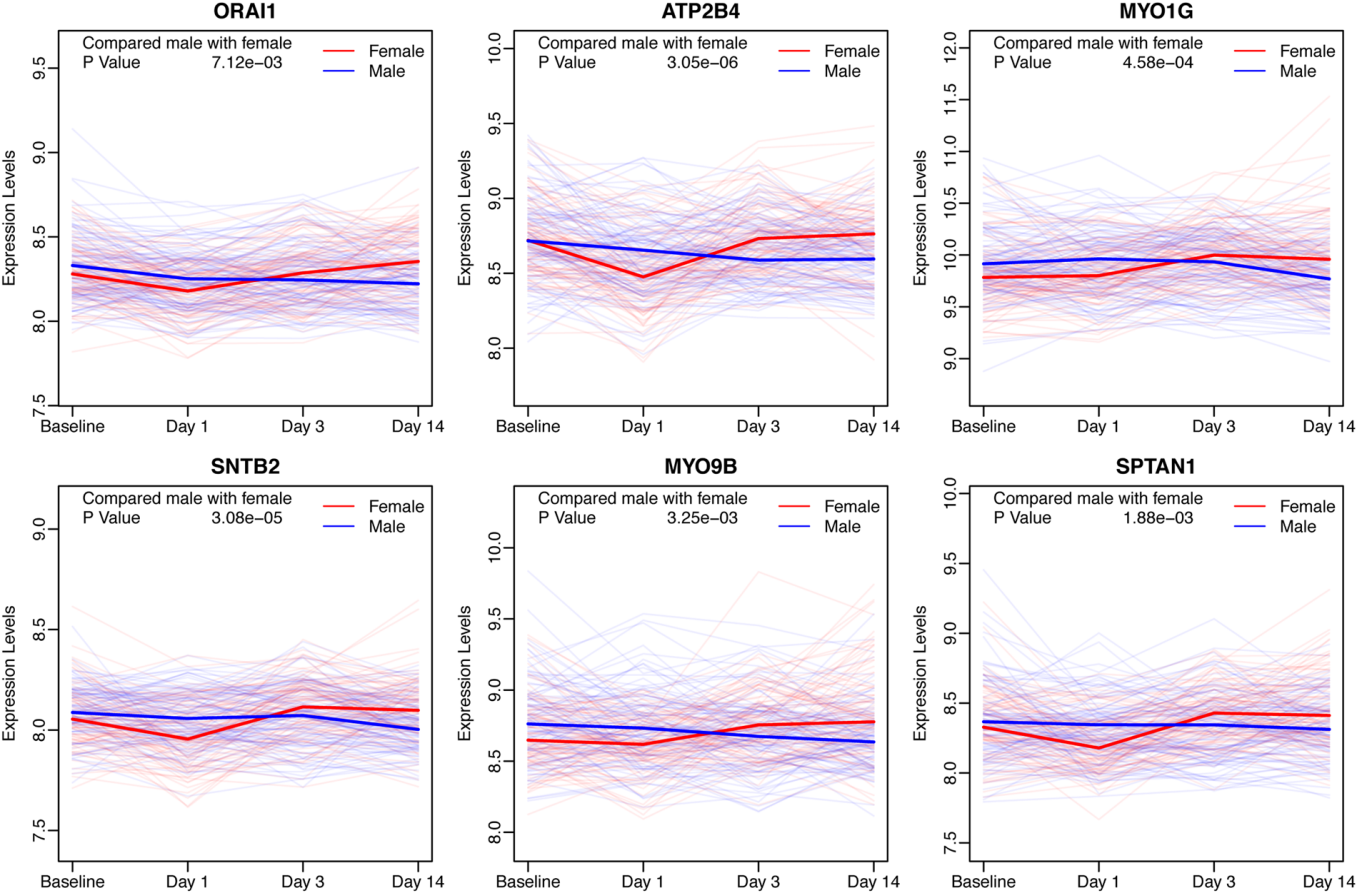
Genes with high expression level variability between females and males in the pathway of calmodulin binding. The gene expression levels of females and males are shown as red and blue, respectively. The solid lines represented the median in the two groups for each gene.

Gene enrichment analysis was carried out for the genes showing variability between females and males (Table 3). The genes highly expressed in females on Day 1 were shown to be associated with the immune response. Unexpectedly, the genes highly expressed in males on Day 1 were shown to be related with calmodulin binding (Fig. 4).

**Table 3:** The GO enrichment for differentially expressed genes between females and males.

	Group	Term	P Value	FDR
Lower in Female	MF	GO:0005516~calmodulin binding	1.18E-05	1.46E-02
	CC	GO:0016020~membrane	1.51E-05	1.85E-02
Lower in Male	CC	GO:0005829~cytosol	1.80E-10	2.28E-07
	BP	GO:0060333~interferon-gamma-mediated signaling pathway	1.76E-08	2.81E-05
	MF	GO:0005515~protein binding	2.40E-07	3.20E-04
	BP	GO:0051607~defense response to virus	3.77E-07	6.02E-04
	BP	GO:0006915~apoptotic process	1.22E-06	1.95E-03
	BP	GO:0006955~immune response	8.41E-06	1.34E-02

**Figure 5:**
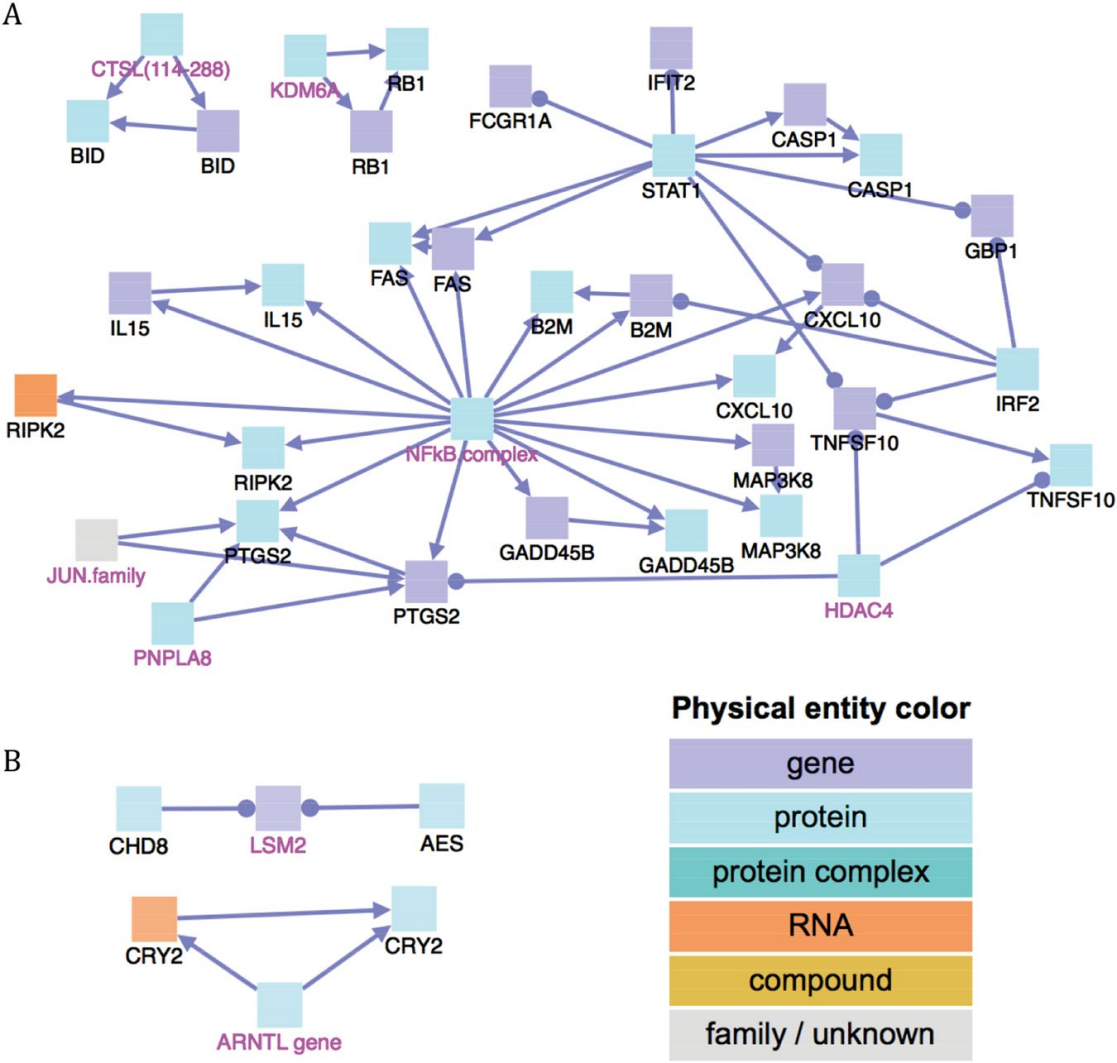
The biological regulatory network for the genes with inter-sex variability. (**A**) Represents the network, which was dominantly regulated by *NF*-*κB* complex, for genes which were expressed more highly in females on Day 1 and (**B**) represents the network for genes that were lower expressed in females on Day 1. The color of the squares depicts the physical entities represented. Black node labels denoted the input genes and magenta node labels denote intermediate nodes. The arrow from protein to gene suggested that the protein activates the gene by regulating the DNA; The arrow from protein to RNA suggested that the protein activates the gene by regulating the RNA.

Finally, ConsensusPathDB-human was utilized to study the biological regulation of these genes with inter-sex variability. The analysis suggested that these genes shared some regulatory elements. For example, though NF-κB complexes did not show differences between sexes, a lot of genes regulated by *NF-κB* did vary. Also, *LSM2*, which was not in the seed node list, is repressed by *CHD8* and *AES* proteins (Fig. 5), indicating *LSM2* might be important in the response to the vaccination.

## Discussion

Our results suggested that the genes activated or repressed after vaccination were related to the immune response, as expected. The GO term enriched for these genes were mainly associated with interferon signaling, involving multiple interferon molecules, instead of other pathways. One interesting observation is that these interferon-related genes were activated within 24 hours after vaccination in females and/or males, although it was reported that type I interferon signaling was inhibited by influenza A virus through modulation of *NF-κB*^18^. These differences could be due to the different types of vaccine used in each study; trivalent inactivated virus or attenuated virus.

A total of 6 genes that were significantly activated with at least 2-fold change after vaccination in both females and males were identified (Table 1). These 6 genes were *WARS*, *STAT1*, *GBP5*, *GBP1*, *EPSTI1 and IFITM3*. There is not a study reporting the relationship of *WARS* and influenza infection, but all other 5 genes are associated with influenza in some aspects. *STAT1* is a transcription factor involved in interferon signaling, that is shared by types I, II, and III interferon. Lee, *et al*.^19^ reported that *STAT1* signaling played a detrimental role in influenza infection by controlling the magnitude of type 17 immune activation. *GBP1*, an interferon-inducible protein, was reported to inhibit the replication of influenza virus by contributing to the host immune response^20^. Results of Feng, *et al*.^21^ revealed that *GBP5* inhibited virus replication through the activation of influenza signaling and pro-inflammatory factors. *IFITM3* also restricted the replication of influenza^22^. The gene expression of *EPSTI1* was significantly increased by adjuvanted vaccine in multiple immune cell types^23^, though the function of it is still unknown.

Another shared profile between females and males is that most of the genes were regulated within 24 hours of administration of the vaccine. Bucasas, *et al*.^17^ also reported that genes involved in antigen presentation and interferon-signaling pathways are observed strongly up-regulated expression in the initial 24 hours after vaccination. Our results suggested that the first 24 hours after influenza vaccination are of great biological importance to the success of the vaccine.

The identified gene profiles further helped to understand the different responses between females and males to vaccination. The first difference was that the magnitude of the response was distinct and that females had stronger immune responses than males. The second difference was that some genes were only activated in females, or only in males. The highly expressed genes in females were enriched in the GO terms and related to the immune response, which was similar to the terms obtained for the shared genes between the females and males. According the biological regulatory network, *NF-κB* complexes played a central role in activating these genes. These genes were critical in order to activate the inflammatory cytokine expression or the innate immune system, so females possibly owned a stronger immune response within post-vaccination Day 1. On the other hand, the lower expressed genes in females were associated with calmodulin binding, which might be a potential pathway involving in the vaccination. These data suggested that genes involved in the immune response were more activated whereas genes involved in the calmodulin binding pathway were more repressed in females than in males within 24 hours. As a result, females may have the advantage to repress the vaccination response within 24 hours by activating the *NF-κB* complex and the type I interferon signaling. Another phenomenon was that most of the differentially expressed genes on Day 1, between males and females, recovered to baseline levels and their expression level were reversed to the opposite phenotype on Day 3 or Day 14 (Table 2). Thus, males may exhibit a stronger response against the vaccines after Day 1 through a more durable response to the vaccination.

Some studies also reported different immunological responses to the virus vaccines between females and males. Considering that the participants analyzed in this study were aged between 18 and 40 and that the impacts of vaccination were also highly age-dependent, only the studies based on adults were regarded as comparable. For example, adult women generated a more robust antibody response following vaccination than men in the US^24^. Meanwhile, we observed that the same dose of influenza vaccine led to a stronger response in females than in males. However, it was reported that the adverse reactions were stronger in females than males^25^, which could be correlated with the stronger response. On the other hand, the likelihood of effective vaccination was unclear, based on the different results of multiple studies^24,26^. So the longer responses to the vaccines in male might also help their outcome of successful vaccination.

Extending from the influenza vaccination, the incidence, morbidity and mortality of seasonal influenza infection showed differences between females and males. For example, it has been reported that the incidence of infection with seasonal influenza viruses was higher in males than females in the United States of America and Spain^5,6^. However, other studies showed the opposite, depending on the regions or populations. The morbidity and mortality were also inconsistent among different studies. Since real influenza infections are affected by multiple factors, including environmental, political, ethnic factors, elucidating the genetic factors is difficult. Some studies based on the mouse models suggested that the possible genetic causes of these differences. Krementsov, *et al*.^27^ reported that the genetic variation in chromosome Y had impacts on the susceptibility to influenza virus in males. Similar genetic variations might also affects the efficacy of the vaccination, but no study was carried out based on this idea. Though some information is known regarding the inter-sex variability through studying the responses to the influenza vaccination, more studies are needed to interpret how the genetic factors affected the incidence, morbidity, and mortality of seasonal influenza infections. These studies will help to produce more effective vaccines or medicines in order to prevent the incidence and mortality caused by influenza.

In conclusion, our results suggested that a higher proportion of genes associated with ‘immune response’ were highly expressed Day 1 post-vaccination in females compared to males, while the genes related to ‘calmodulin binding’ were the opposite. This suggested that females might generate stronger immune responses to seasonal influenza vaccines within the first 24 hours. However, most of these genes with variability between males and females were found had the opposite expression levels on Day 3 or Day 14, which suggested that the influenza vaccines provided longer immunity in adult male than in adult female.

## Materials and Methods

### Data Preprocessing

The un-normalized data from two datasets, GSE48023 and GSE48018, were extracted from Gene Expression Omnibus (GEO), since the raw Illumina beadchip files were unavailable. These two datasets were generated by Belmont Lab, Baylor College of Medicine^1,17^. The GSE48023 and GSE48018 contain whole-blood RNA samples from 128 healthy female and 119 healthy male volunteers ages 19–41 years, respectively. The probes used only in females or males were removed first. The data were normalized using limma package in R software to make the columns of a matrix have the same quantiles^28^. The batch adjustment between females and males was completed using R package sva^29^. ComBat function was applied to adjust for known batches using an empirical Bayesian framework^30^, so the batch differences between females and males could be minimized. The expression levels of the probes were mapped to the genes according to the table from platform GPL6947 and GPL10558 for males and females, respectively. If several probes were mapped to one gene, the median was used to represent the expression level of that gene.

### Genes Responding to Influenza Vaccination

The genes differentially expressed after vaccination were identified using limma. Linear models and empirical Bayes methods were performed as previously described to assess the differential expressions between genes^31^. Next, the contrasts were built between each time point, Day 1, Day 3, and Day 14, and the baseline for females and males, separately. The combined p-values were adjusted using FDR and the differentially expressed genes were defined as the FDR (false discovery rate) lower than 0.01.

### Genes Involved in Inter-Sex Variability

Similar models were applied to identify genes that were differentially expressed between females and males, except that the contrasts were built between females and males from the same time series. The genes that were differentially expressed between females and males after vaccination were identified with a combined p-value, after FDR adjustment, lower than 0.01. The differentially expressed genes in the baseline category between females and males were identified using the same cutoff. After that, the final list of genes showing different responses to vaccine were obtained by removing these originally sex-specific genes from the differentially expressed gene list^32,33^.

### Biological Function Analysis

The GO terms were enriched using DAVID Bioinformatics Resources v6.8^34,35^ for higher or lower expressed genes in females vaccinated with influenza compared with males. The p-values were adjusted using FDR and 0.05 was used as the cutoff. ConsensusPathDB-human was used to study the biological regulation of these genes.

## References

[1] Franco LM (2013). Integrative genomic analysis of the human immune response to influenza vaccination. Elife.

[2] Smith W, Andrewes CH, Laidlaw PP (1995). A Virus Obtained from Influenza Patients. Lancet.

[3] Trammell RA, Toth LA (2008). Genetic susceptibility and resistance to influenza infection and disease in humans and mice. Expert Rev Mol Diagn.

[4] Organization, W. H. Sex, gender and influenza. *Geneva World Health Organisation* (2010).

[5] Larrauri A, De MS (2007). Characterisation of swabbing for virological analysis in the Spanish Influenza Sentinel Surveillance System during four influenza seasons in the period 2002–2006. Eurosurveillance: bulletin europeen sur les maladies transmissibles = European communicable disease bulletin.

[6] Szilagyi PG (2008). Influenza vaccine effectiveness among children 6 to 59 months of age during 2 influenza seasons: a case-cohort study. Arch Pediatr Adolesc Med.

[7] Pulcini C, Massin S, Launay O, Verger P (2013). Factors associated with vaccination for hepatitis B, pertussis, seasonal and pandemic influenza among French general practitioners: a 2010 survey. Vaccine.

[8] Klein SL, Jedlicka A, Pekosz A (2010). The Xs and Y of immune responses to viral vaccines. The Lancet. Infectious diseases.

[9] Lorenzo ME (2011). Antibody responses and cross protection against lethal influenza A viruses differ between the sexes in C57BL/6 mice. Vaccine.

[10] Wang XL (2015). Age and Sex Differences in Rates of Influenza-Associated Hospitalizations in Hong Kong. Am J Epidemiol.

[11] Quandelacy TM, Viboud C, Charu V, Lipsitch M, Goldstein E (2014). Age- and sex-related risk factors for influenza-associated mortality in the United States between 1997–2007. Am J Epidemiol.

[12] Fink AL, Klein SL (2015). Sex and Gender Impact Immune Responses to Vaccines Among the Elderly. Physiology.

[13] Mosby LG, Rasmussen SA, Jamieson DJ (2011). 2009 pandemic influenza A (H1N1) in pregnancy: a systematic review of the literature. American journal of obstetrics and gynecology.

[14] Jamieson DJ (2009). H1N1 2009 influenza virus infection during pregnancy in the USA. Lancet.

[15] Neuzil KM, Reed GW, Mitchel EF, Simonsen L, Griffin MR (1998). Impact of influenza on acute cardiopulmonary hospitalizations in pregnant women. Am J Epidemiol.

[16] Creanga AA (2010). Severity of 2009 pandemic influenza A (H1N1) virus infection in pregnant women. Obstetrics and gynecology.

[17] Bucasas KL (2011). Early patterns of gene expression correlate with the humoral immune response to influenza vaccination in humans. J Infect Dis.

[18] Pauli EK (2008). Influenza A virus inhibits type I IFN signaling via NF-kappaB-dependent induction of SOCS-3 expression. Plos Pathog.

[19] Lee B (2017). STAT1 Is Required for Suppression of Type 17 Immunity during Influenza and Bacterial Superinfection. ImmunoHorizons.

[20] Zhu Z (2013). Nonstructural protein 1 of influenza A virus interacts with human guanylate-binding protein 1 to antagonize antiviral activity. Plos One.

[21] Feng J (2017). Inducible GBP5 Mediates the Antiviral Response via Interferon-Related Pathways during Influenza A Virus Infection. J Innate Immun.

[22] Everitt AR (2012). IFITM3 restricts the morbidity and mortality associated with influenza. Nature.

[23] Howard LM (2017). Cell-Based Systems Biology Analysis of Human AS03-Adjuvanted H5N1 Avian Influenza Vaccine Responses: A Phase I Randomized Controlled Trial. Plos One.

[24] Engler RJ (2008). Half- vs full-dose trivalent inactivated influenza vaccine (2004–2005): age, dose, and sex effects on immune responses. Arch Intern Med.

[25] Endrich MM, Blank PR, Szucs TD (2009). Influenza vaccination uptake and socioeconomic determinants in 11 European countries. Vaccine.

[26] Beyer WEP, Palache AM, Kerstens R, Masurel N (1996). Gender differences in local and systemic reactions to inactivated influenza vaccine, established by a meta-analysis of fourteen independent studies. European Journal of Clinical Microbiology & Infectious Diseases.

[27] Krementsov DN (2017). Genetic variation in chromosome Y regulates susceptibility to influenza A virus infection. Proc Natl Acad Sci USA.

[28] Ritchie ME (2015). limma powers differential expression analyses for RNA-sequencing and microarray studies. Nucleic Acids Res.

[29] Leek JT, Johnson WE, Parker HS, Jaffe AE, Storey JD (2012). The sva package for removing batch effects and other unwanted variation in high-throughput experiments. Bioinformatics.

[30] Johnson WE, Li C, Rabinovic A (2007). Adjusting batch effects in microarray expression data using empirical Bayes methods. Biostatistics.

[31] Wen F (2017). A meta-analysis of transcriptomic characterization revealed extracellular matrix pathway involved in the H5N1 and H7N9 infections. Oncotarget.

[32] Herwig R, Hardt C, Lienhard M, Kamburov A (2016). Analyzing and interpreting genome data at the network level with ConsensusPathDB. Nat Protoc.

[33] Kamburov A, Stelzl U, Lehrach H, Herwig R (2013). The ConsensusPathDB interaction database: 2013 update. Nucleic Acids Res.

[34] Huang da W, Sherman BT, Lempicki RA (2009). Bioinformatics enrichment tools: paths toward the comprehensive functional analysis of large gene lists. Nucleic Acids Res.

[35] Huang da W, Sherman BT, Lempicki RA (2009). Systematic and integrative analysis of large gene lists using DAVID bioinformatics resources. Nat Protoc.

